# Gene co-expression network analysis reveals key potential gene modules in utero-vaginal junction associated with duration of fertility trait of breeder hens

**DOI:** 10.1038/s41598-019-50148-6

**Published:** 2019-09-25

**Authors:** Lantao Gu, Ruoxi Jing, Yanzhang Gong, Mei Yu, Abdelmotaleb Elokil, Shijun Li

**Affiliations:** 1 Key Laboratory of Agricultural Animal Genetics, Breeding and Reproduction of Ministry of Education, College of Animal Science and Technology, Huazhong Agricultural University, Wuhan, Hubei China; 2 Guilin Medical University, Guilin, Guangxi China; 3 Department of Animal Production, Faculty of Agriculture, Benha University, Moshtohor, Egypt

**Keywords:** Reporter genes, Agricultural genetics

## Abstract

The number of days (DN) when hens lay fertile eggs as well as the number of fertile eggs (FN) were produced after a single artificial insemination (AI), including the two duration of fertility (DF) traits. Indeed, they are the key production performance that associates with the production cost of hatching egg when its determination the interval between successive artificial inseminations. However, the relevant genes response for regulating the DF has not been uncovered yet. Therefore, we performed a weighted gene co-expression network analysis (WGCNA) to investigate the insight into co-expression gene modules on DF process in hens. The total mRNA was extracted from the utero-vaginal junction (UVJ, with the sperm storage function in hen’s oviduct which is the biological basis for DF) of 20 hens with several levels of DF traits, and performed transcriptome sequences of mRNA. As a result, three co-expression gene modules were identified to be highly correlated with DF traits. Moreover, the expression changes of top 5 hub genes in each module with DF traits were further confirmed in other 20 hens by RT-PCR. These findings highlighted the co-expression modules and their affiliated genes as playing important roles in the regulation of DF traits.

## Introduction

The avian females have the ability to store sperm in their reproductive tracts following the natural copulation or AI for days or weeks (depending on the species), locating utero-vaginal junction (UVJ). Thus, its produce a series of fertile eggs following a single copulation event or artificial insemination (AI)^1^. This period of sustained fertility is generally identified as the duration of fertility (DF, days number between insemination and the last fertile egg)^2^. The long or short DF are identified the frequency of AI because any reduction in this frequency without loss of fertility directly reduces the cost of producing hatching eggs, thus to cut down the number of breeder males and labor costs associated with AI^3^. Meanwhile, it could alleviate the stress and suffering of hens from frequent AI. Therefore, study on the molecular mechanism that regulating DF traits can not only develop a clear understanding of this process, but also can improve the economic efficiency for production the hatching eggs. In mature hens’ oviduct, many specialized simple tubulars invaginate the utero-vaginal junction (UVJ) mucosal folds^4^. These tubulars protects a long-time sperm storage function, thus were referred to as sperm storage tubules (SSTs). The utero-vaginal sperm storage tubules functions as a sperm reservoir in hen’s oviduct^5^. Here, sperms are ultimately released and upward transport to the infundibulum for ova fertilization. This function gives hens a senseful capacity that hens could lay fertile eggs for days or weeks without frequently insemination^6,7^. Therefore, investigation the relationship of gene expression in UVJ to the duration of fertility traits could add our understanding about the molecular regulatory mechanism participating in the duration of fertility process.

In this study, RNA sequenced and WGCNA presented the key potential gene co-expression modules associated with breeder hens’ duration of fertility traits and their possible implications in duration of fertility process.

## Material and Methods

### Ethics statement

The experimental procedures used in this study met the guidelines of the Care and Use of Laboratory Animals of the Standing Committee of Hubei People’s Congress (No. 5) and approved by the Biological Studies Animal Care Committee of Hubei Province, P.R. China, and the Ethics Committee of Huazhong Agricultural University, PR China. All efforts were made to minimize the animal suffering.

### Animal management and trait measurement

A total of 450 healthy Jinghong breeder hens (parents) at 30 weeks old were obtained from the poultry farm of Huadu Yukou Poultry Industry Co. Ltd (Beijing, China). All birds were raised in individual cages, kept in identical light/dark cycles and had ad libitum access to healthy water and a commercial diet until the end of the experiment. All hens were artificially inseminated once with 2.00 × 10^8^ sperms issued from pooled ejaculates. To prevent any undesirable effects of the interval between inseminations and oviposition on subsequent fertility, insemination was done identically in afternoon. Eggs were collected and marked daily for 20 days after AI and checked the fertility by candling eggs after 10 days from incubation (dead embryos were considered as fertile). Two duration of fertility traits DN and FN were measured in terms (DN = the number of days post-insemination until the last fertile egg was produced; and FN = the number of fertile eggs were produced after a single AI). DN and FN were expressed as average of three measurements^8^.

### RNA sequencing and data preprocessing

A total of 21 hens with their phenotypes distributed throughout duration of fertility traits were sorted out from the experimental population (Table S2). On the 13^th^ day after AI, these hens were euthanized by decapitation under anesthesia. UVJ tissues were dissected immediately and removed adhering connective tissues^9^. Total RNA was isolated using Trizol reagent (Invitrogen, Foster City, CA, USA), following the recommended manufacturers protocol. The quality and quantity of RNA samples were detected by 1.0% agarose gel electrophoresis and absorbance optical density (OD) at a 260/280 nm ratio, respectively^8^. Pair-end 150-bp reads were generated on a Hiseq 2500 sequencing machine at the Shanghai biotechnology corporation. Additionally, aligned to the chicken reference genome by HISAT2(version: 2.0.9). A raw counts data was obtained from the HTSEQ-count (version: 3.0.8) Soft-ware and the genes with row counts less than 15 in all samples were removed^10,11^. These steps finally resulted in more than 17000 genes to infer next study. Then the raw counts data were processed and normalized by edgeR Soft-ware to generate an FPKM expression matrix^12^. Sample clustering was performed using the hierarchical average linkage clustering function implemented in the WGCNA R package to identify possible outliers. One sample (Sample ID: 7) was identified as an outlier and thus excluded from the final dataset (Supplementary File 1, Fig. S1).

### Co-expression network construction and meta-modules detection

Networks were formed following the protocols of WGCNA^13^. Briefly, a similarity matrix was constructed using the Pearson correlation coefficients created between the normalized expression levels of the input transcripts. By raising the absolute value of the Pearson correlation coefficients to a power of 9, we could get a scale-free topology index above 0.9, achieving a network with few, large correlations at the expense of lowly correlated transcripts. This allows for the fewer, highly connected and biologically relevant hub genes. An adjacency network was then created using topological overlap measure (TOM, a measure of neighborhood connectivity between two genes)^14^. To create modules, the adjacency network was converted into a dissimilarity measure (1-TOM) and clustered using flashClust (a hierarchical clustering function). Cluster branches were cut to identify modules. Module size was set to a minimum of 30 transcripts and modules with a 75% similarity were merged using dynamic tree cutting, resulting in 54 modules^10^.

Module eigengene (Me) was defined as the first principal component of a given module, which represented the gene expression profiles in a module. Generally, a module with high correlation between Me and DF traits could be meta-module that associated with DF traits. Hub genes tends to be in the center of a network, highly connected with other genes and hence of high functional significance. The intramodular connectivity (K.in) was calculated as the summation of adjacency performed over all genes in a network. A gene with high K.in means that this gene is the most important elements of modules associated with relevant trait. Therefore, a gene with high K.in in a module was hub gene.

### RNA expression analyses by quantitative RT-PCR

At last, total RNA from UVJ tissues of the 20 hens (one of 21 hens was found to be outliers) were isolated. cDNA was synthesized using TransScript One-Step gDNA Removal and cDNA Synthesis SuperMix (Trans, Beijing) as recommended by the manufacturer. The resulting cDNA was subjected to real-time quantitative PCR with SYBR Green Mix (QIAGEN, Germany) and specific primer sets (Table S4) in accordance with the manufacturer instructions. PCR products were verified by melting curve analysis. The relative quantification expression was calculated using the delta-delta Ct method with each gene normalized to GAPDH.

## Results

### RNA sequencing and weighted gene co-expression network construction

Respectively, the 450 hens’ duration of fertility traits were 14.31 ± 2.38 days (Coefficient of variation:16.66%; Percentiles 2.5–97.5%: 8.35–18.00) for DN, and 10.66 ± 2.53 eggs (Coefficient of variation: 23.71%; Percentiles 2.5–97.5%: 4.67–15.00) for FN. The Correlation coefficients of FN and DN phenotypic values was 0.53. Thereafter, the duration of fertility of hens were divided into 10 levels according to the DN and FN respectively. To make sure that there are at least one sample in each level, a total of 21 hens with their duration of fertility traits distributed throughout ten levels were sorted out for RNA sequencing (Tables S1 and S2). More than 40 million sequencing reads were generated for each sample, and more than 70% of these reads were exactly mapped onto the chicken reference genome (Table S3). After filter genes with row counts less than 15 in all samples, we finally obtained the expression profiling of more than 17 000 genes in UVJ. But one sample (Sample ID: 7) was foung to be an outlier and thus excluded from the final dataset.

**Figure 1:**
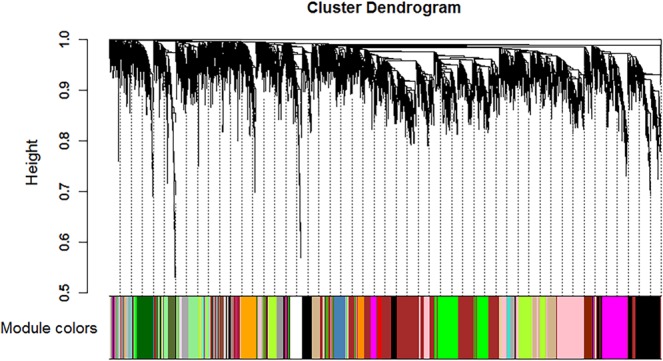
Identification of gene co-expression modules in utero-vaginal junction of hens. Module size was set to a minimum of 30 transcripts and modules with a 75% similarity were merged using dynamic tree cutting, resulting in 54 color-coded modules.

After preprocessing the RNA sequencing data, we applied the WGCNA package to compile the co-expression network. Keeping to the scale-free topology criterion, β = 9 was considered in this study. Following dynamic tree cut, the topological overlap clustering dendrogram identified 54 distinct gene modules, as can be seen from the color-band underneath the cluster tree (Fig. 1). The gray module consisted of genes that do not group into any specific module.

### Identification of meta-modules associated with duration of fertility

**Figure 2:**
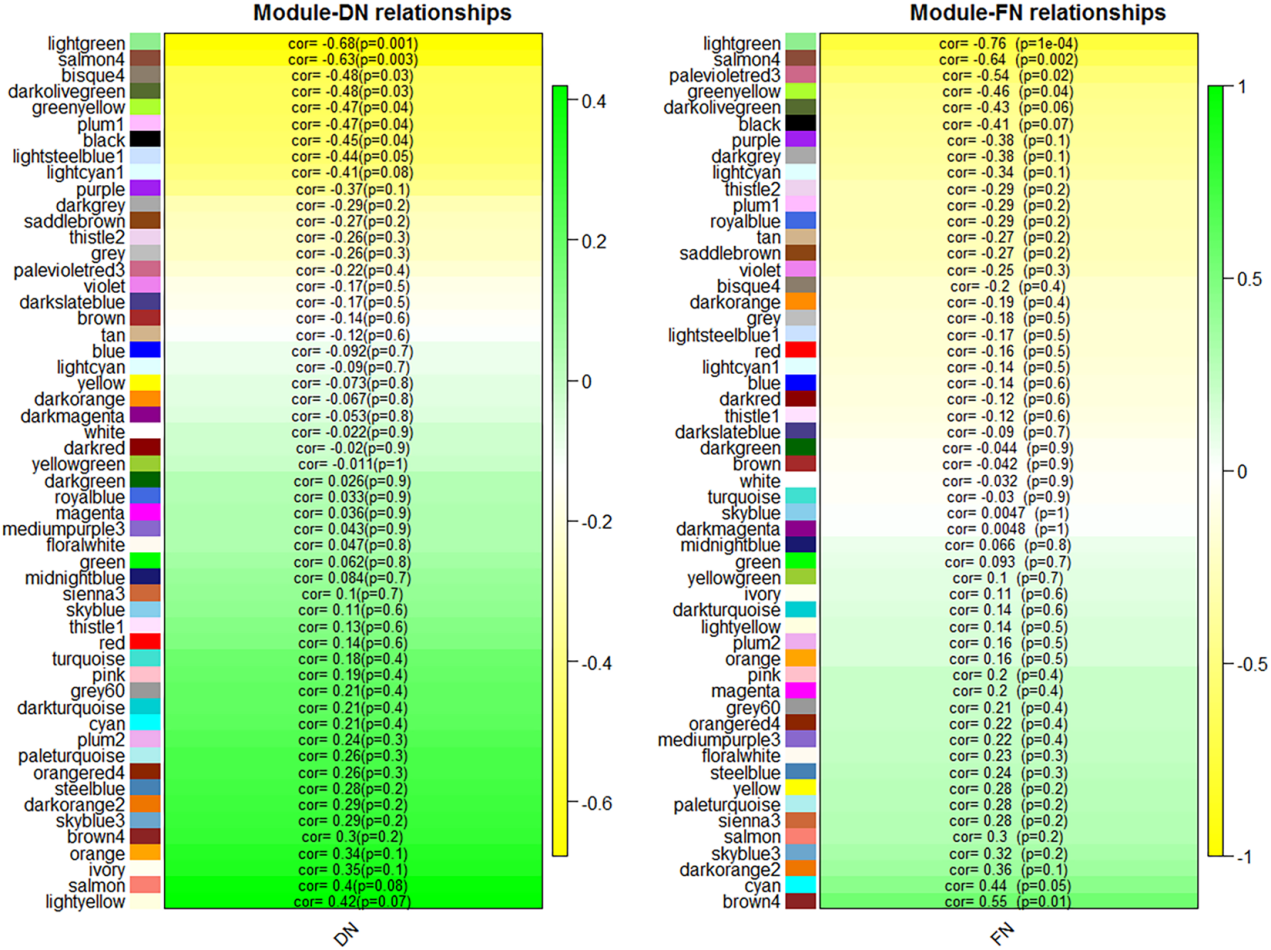
Heatmap reporting correlations (and corresponding p-values) between modules and duration of fertility traits. DN = the number of days post-insemination until the last fertile egg was produced; and FN = the number of fertile eggs were produced after a single AI.

**Figure 3:**
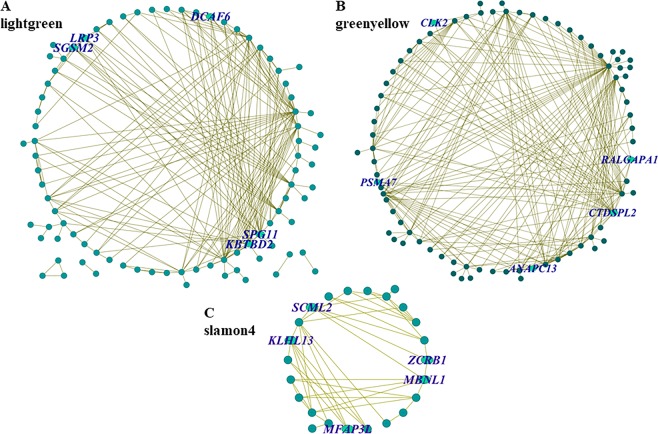
Interaction of gene co-expression network of the duration of fertility related modules. The modules (**A**: lightgreen, **B**: greenyellow, **C**: slamon4) was visualized using Cytoscape 3.0 software. Every node represents a gene, and the nodes labeled by gene symbols were the top five hub genes as ranked by intra-module connectivity.

To identify co-expression modules associated with duration of fertility, we assessed the relationship of duration of fertility traits DN and FN with the module eigengene (Me). Two modules (lightgreen and salmon4) showed strong association with DN (*P* < 0.01) while five modules (bisque4, darkolivegreen, greenyellow, plum1 and black) showed association with DN (*P* < 0.05). The two modules (lightgreen and salmon4) also showed strong association with FN (*P* < 0.01), and three modules (palevioletred3, greenyellow and brown4) were showed associated with FN (*P* < 0.05). Overall, significant correlations of module eigengene with DN and FN both were observed in three modules (lightgreen, salmon4 and greenyellow), thereafter named duration of fertility modules (Fig. 2). The visualized the connectivity patterns and hub genes of the meta-modules were presented in Fig. 3.

### Validation of hub genes

**Figure 4:**
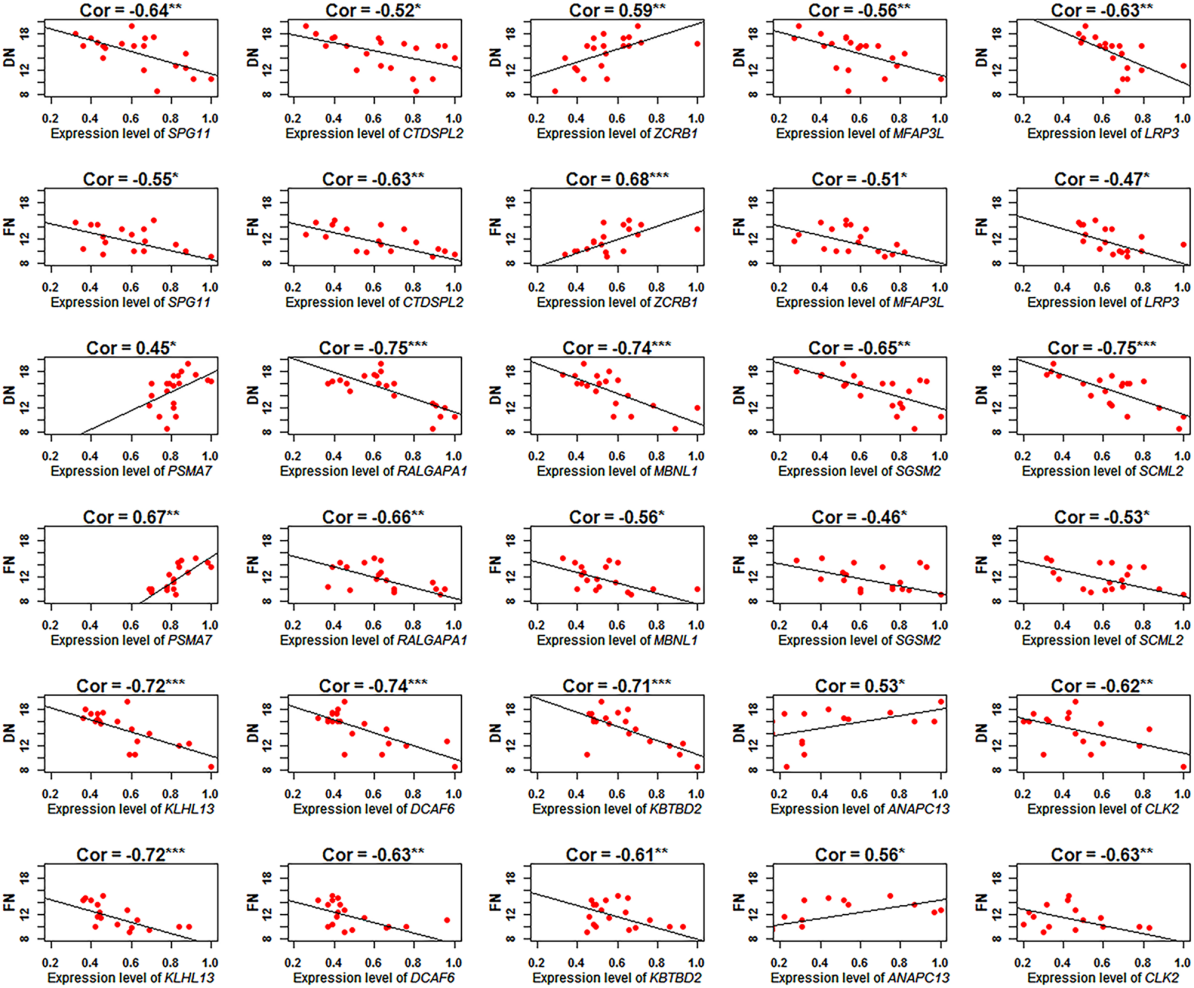
The hub gene expression changes correlated with duration of fertility traits DN and FN. DN = the number of days post-insemination until the last fertile egg was produced; and FN = the number of fertile eggs were produced after a single AI. *P < 0.05, **P < 0.01, ***P < 0.001.

We validated the relative expression levels of hub genes from the three duration of fertility modules using RT-PCR (Fig. 4). The results were consistent with those obtained by RNA-seq, which the expression levels of 12 genes (*SPG11*, *CTDSPL2*, *MFAP3L*, *LRP3*, *RALGAPA1*, *MBNL1*, *SGSM2*, *SCML2*, *KLHL13*, *DCAF6*, *KBTBD2* and *CLK2*) were all significantly down-regulated in the UVJ correlated with duration of fertility traits DN or FN, and the expression levels of 3 genes (*ZCRB1*, *PSMA7* and *ANAPC13*) were all significantly up-regulated in the UVJ correlated with duration of fertility traits.

## Discussion

In the present study, we performed RNA sequencing and a systematic WGCNA to gain a better understanding of the transcriptomic changes in UVJ tissues of hens with duration of fertility trait on a genome-wide scale. Our findings revealed that fifty-four co-expression gene modules were discovered in UVJ of hens and three modules (named light-green, salmon4 and green-yellow) were found significantly associated with duration of fertility traits in this study. It is believed that modules are co-expressed gene clusters with integrated function in biological process. Therefore, functional modules can facilitate the finding of hub genes that are key drivers of consistent biological process^15^. To sum up, the duration of fertility was a complex and systematic process: surviving sperms from the UVJ environment and immunological response, retaining sperms in the SST, activating and taxiing sperm towards oocytes, and removing non-fertilizing sperm^16,17^. The investigation of these modules revealed novel hub genes and pathways that are likely biologically relevant to these events. The green-yellow module consisted of 351 genes, and the top five hub genes were *CTDSPL2*, *RALGAPA1*, *CLK2*, *PSMA7* and *ANAPC13*. These genes (*ANAPC13*, *PSMA7 and CLK2*) have been reported to mainly involving adaptive immune and play an important regulatory mechanism required for promoting cell survival^18–21^. In previous study, it is reported that the female sperm allergy is an important cause leading to the problem of sperm survival, this allergy was present in the hens with short duration of fertility^22,23^. It indicated that the necessary of immune privilege for sperms residing and surviving in SSTs. Along these lines, these genes driving the adaptive immune and cell survival might response for this event. The rest, *ANAPC13* participated in progesterone-mediated oocyte maturation^24^, similarly the *CTDSPL2* has been found to participate in gene regulation during erythroid maturation^25^, and the mRNA level of *RALGAPA1* was reported to specifically associated with total egg number at 500 d of age or egg rate after the first egg^26^. They also seem to be associated with duration of fertility.

The slamon4 module consisted of 40 genes, and the top five hub gene including *MBNL1*, *MFAP3L*, *ZCRB1*, *KLHL13* and *SCML2*. Among these genes, the *MBNL1* and *ZCRB1* both mediate the wide range of tissue-specific RNA processing steps^27^. Particularly, *MBNL1* was reported to negatively regulates *TGFβ3* production autocrine^28^, which coincide to the association of the expression level of *TGFβ3* in UVJ with duration of fertility^8^. Otherwise, the *KLHL13* was reported to participated in adaptive immune system, while the *SCML2* was involved in transcriptional regulation^29^ and the *MFAP3L* was reported to participate in the nuclear signaling of EGFR which involving angiogenesis. We have known that the SST was specialized simple tubular invaginations of the utero-vaginal junction (UVJ) mucous epithelium, and its tissue morphology is like the blood vessels. The genesis of SST might involve this process.

The light-green module consisted of 228 genes, and the top five hub gene including *KBTBD2*, *SPG11*, *DCAF6*, *SGSM2* and *LRP3*. These genes show a mainly biological function of material transport and metabolism. Such as *SGSM2* and *SPG11* was reported to participated in intercellular vesicle transport^30^. The *LRP3* plays a role in endocytosis^31^. The *KBTBD2* participated in lip metabolism^32^, and the *DCAF6* show effects on protein ubiquitination and degradation^33^. These processes might be mediated the supplication of nutrients to the sperm, removing of waste products of sperm metabolism, and the phagocytosis of non-fertilizing sperm.

In conclusion, we found three gene co-expression network and their affiliated hub genes that are associated with duration of fertility traits. Although interpretation of the results in the present study is limited and more surveys will be needed to determine the physiological functions of these genes, it contributes to the understanding of gene regulatory mechanism on duration of fertility.

## Supplementary information

supporting information file

supporting dataset
