# Supplementary material for: Gene co-expression network analysis reveals key potential gene modules in utero-vaginal junction associated with duration of fertility trait of breeder hens
Source: Sci Rep. 2019 Sep 25;9:13860. doi: 10.1038/s41598-019-50148-6 (PMC6761187; doi:10.1038/s41598-019-50148-6)
Supplement: Supplementary file 1 — supporting information file [file 41598_2019_50148_MOESM1_ESM.pdf]

Gene co-expression network analysis reveals key potential gene modules in utero-vaginal junction associated with duration of fertility trait of breeder hens

Lantao Gu [1, 2], Ruoxi Jing [1], Yanzhang Gong [1], Mei Yu [1], Abdelmotaleb Elokil [1, 3], Shijun Li [1 *]

1 Key Laboratory of Agricultural Animal Genetics, Breeding and Reproduction of Ministry of Education, College of Animal Science and Technology, Huazhong Agricultural University, Wuhan, Hubei, China.

2 Guilin Medical University, Guilin, Guangxi, China.

3 Department of Animal Production, Faculty of Agriculture, Benha University, Moshtohor, Egypt.

*Corresponding author: Shijun Li, lishijun@mail.hzau.edu.cn

Table S1. the duration of fertility traits of hens used for RNA sequence.

Sample ID	DN	FN
**1**	11.00±1.00	9.00±1.00
**2**	12.00±1.00	8.33±1.53
**3**	12.67±1.53	11.00±1.00
**4**	14.00±1.00	9.33±1.15
**5**	16.00±0.00	10.33±1.15
**6**	14.67±1.53	9.67±0.58
**7**	12.33±1.53	10.00±1.73
**8**	10.33±1.15	6.00±1.73
**9**	9.00±1.00	5.67±1.15
**10**	6.33±1.53	3.67±1.15
**11**	16.00±1.00	14.33±1.15
**12**	16.00±1.00	12.33±0.58
**13**	16.00±2.00	13.67±0.58
**14**	15.67±1.53	13.67±0.58
**15**	17.33±1.53	15.00±1.00
**16**	17.33±1.15	14.33±2.31
**17**	19.33±0.58	12.67±0.58
**18**	18.00±1.00	14.67±0.58
**19**	15.67±1.53	11.33±0.58
**20**	16.00±0.00	7.67±1.53
**21**	17.33±1.53	11.67±2.08

DN = the number of days post-insemination until the last fertile egg was produced; and FN = the number of fertile eggs were produced after a single AI. Values are present as Mean±SD of three measurement.

Table S2. Ten levels of duration of fertility traits and the relevant samples in each level.

DN	Samples ID	FN	Samples ID
<8	10	< 4.5	10
8 - 9.3	9	4.5 - 5.8	9
9.3 - 10.6	8	5.8 - 7.1	8
10.6 - 11.9	1	7.1 - 8.4	2
11.9 - 13.2	2; 7; 3;	8.4 - 9.7	1; 4; 6
13.2 - 14.5	4	9.7 - 11	7; 5; 3
14.5 - 15.8	6; 19; 14	11 - 12.3	19; 21
15.8 - 17.1	20; 5; 12; 13; 11	12.3 - 13.6	12; 17
17.1 - 18.4	21; 16; 15; 18	13.6 - 14.9	14; 13; 11; 16; 18
> 18.4	17	> 14.9	15

DN = the number of days post-insemination until the last fertile egg was produced; and FN = the number of fertile eggs were produced after a single AI. The hens' duration of fertility traits DN and FN are normally distributed, and 95% hens were ranged from 8.35-18.00 and 4.67-15.00 respectively. Therefore, the hens were divided into 10 levels according to the DN, including <8, 8-9.3, 9.3-10.6, 10.6-11.9, 11.9-13.2, 13.2-14.5, 14.5-15.8, 15.8-17.1, 17.1-18.4, >18.4. And according to the FN, they were also divided into 10 levels: <4.5, 4.5-5.8, 5.8-7.1, 7.1-8.4, 8.4-9.7, 9.7-11, 11-12.3, 12.3-13.6, 13.6-14.9, >14.9. Twenty-one hens were selected to making sure that there are at least one sample in each level were used for RNA sequencing.

Table S3. the RNA sequence result after quality control.

Sample ID	Raw reads	Clean reads	Clean ratio	Mapped reads	Mapping ratio
**1**	41 130 278	34 884 196	84.81%	25 705 055	73.69%
**2**	43 892 552	39 684 001	90.41%	28 888 083	72.80%
**3**	43 961 908	35 884 698	81.63%	26 732 395	74.50%
**4**	41 283 652	33 647 366	81.50%	24 279 224	72.16%
**5**	40 136 168	33 113 619	82.50%	23 923 796	72.25%
**6**	40 088 882	31 612 063	78.85%	23 227 838	73.48%
**7**	43 539 586	40 121 186	92.15%	29 153 043	72.66%
**8**	46 562 544	42 057 498	90.32%	30 903 366	73.48%
**9**	43 885 076	39 269 096	89.48%	28 946 760	73.71%
**10**	46 951 108	41 988 103	89.43%	31 467 231	74.94%
**11**	41 835 408	34 653 595	82.83%	25 247 392	72.86%
**12**	45 608 594	41 205 537	90.35%	30 132 184	73.13%
**13**	49 674 102	43 713 435	88.00%	31 818 985	72.79%
**14**	44 831 188	37 346 451	83.30%	27 328 050	73.17%
**15**	43 294 774	35 624 162	82.28%	25 746 064	72.27%
**16**	42 552 386	33 843 528	79.53%	24 736 750	73.09%
**17**	46 773 856	38 270 144	81.82%	28 077 143	73.37%
**18**	44 089 100	35 279 337	80.02%	26 004 719	73.71%
**19**	41 473 644	36 657 696	88.39%	27 066 117	73.83%
**20**	46 392 040	42 170 024	90.90%	30 958 973	73.41%
**21**	43 113 222	39 916 485	92.59%	29 530 245	73.98%

Clean ratio = Clean reads/Raw reads; Mapping ratio=Mapped reads/All reads. Reference genomes: Gallus_gallus.Gallus_gallus-5.0.

Table S4. Primer pairs used for RT-PCR.

Primer pairs	Sequence (5'-3')
F- KBTBD2	CGCCAGCCTAACATGAACAC
R- KBTBD2	TCAACTTCACGCAGAAAGCA
F- DCAF6	GGAAGAGTTGGAGACTTTGA
R- DCAF6	AAATGGATGAGGCTGAAGAC
F- LRP3	CAAGCTGCCTACTTATATTC
R- LRP3	GCTCATTGGGTTACATAGTC
F- MBNL1	GTAAGAATGTCAGTCATCGT
R- MBNL1	CCATAGCAACAACTTAACTC
F- MFAP3L	AGGACGCTTGAACTTAACAC
R- MFAP3L	ATGGCCTTCTCAATCACAAC
F- ZCRB1	CTTCTTGCAGGTAGTATCTT
R- ZCRB1	TACAACCCCATGCAGAGAAG
F- KLHL13	ATAGGTGGATAAGGGCTAGA
R- KLHL13	CAGAGGACACGTTATGCAAG
F- CLK2	TCTGCATGTCCATACTGTAA
R- CLK2	CACGGAAACAGAGAAGCTAC
F-ANAPC13	AAGTCATAGGGAAGCACCAG
R-ANAPC13	CGATGGTGTGAAGGAATCAG
F1- SPG11	GTTCAGTCATTAGTGCCGTT
R1- SPG11	AGTCCAGTCGTTCCATTACA
F1-SCML2	CGATAGAGGAAGTAATGTGT
R1-SCML2	CAGCTTCAGTCCCATGTATT
F1- RALGAPA1	TGCAAGGGATGGAATTACAC
R1- RALGAPA1	AGCAGGGATGTTGAGTGAGA
F1- PSMA7	GAAGCCATTGAAACAGATGA
R1- PSMA7	ATCTCTTCTCATAACAGCCA
F1-CTDSPL2	TATAGAAAGCTGGTTCATGG
R1-CTDSPL2	CGAAATCTGTCTCTGATGTG
F1- SGSM2	CCAACATGCGGTCTCTAATC
R1- SGSM2	ATCCTCGTACAACAATTCTC

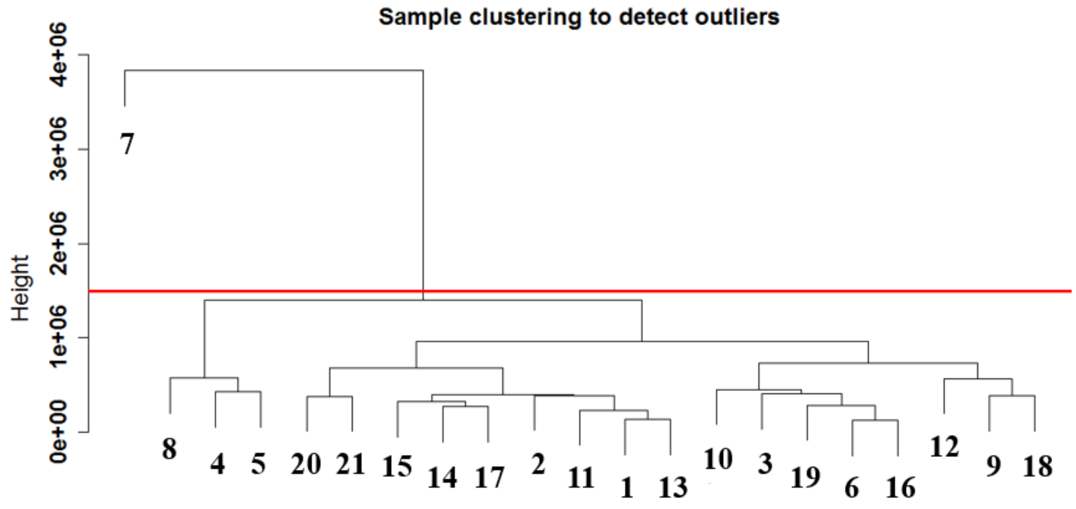

Figure S1 Clustering dendrogram of samples based on their Euclidean distance. The red line means the threshold indicating the outliers.

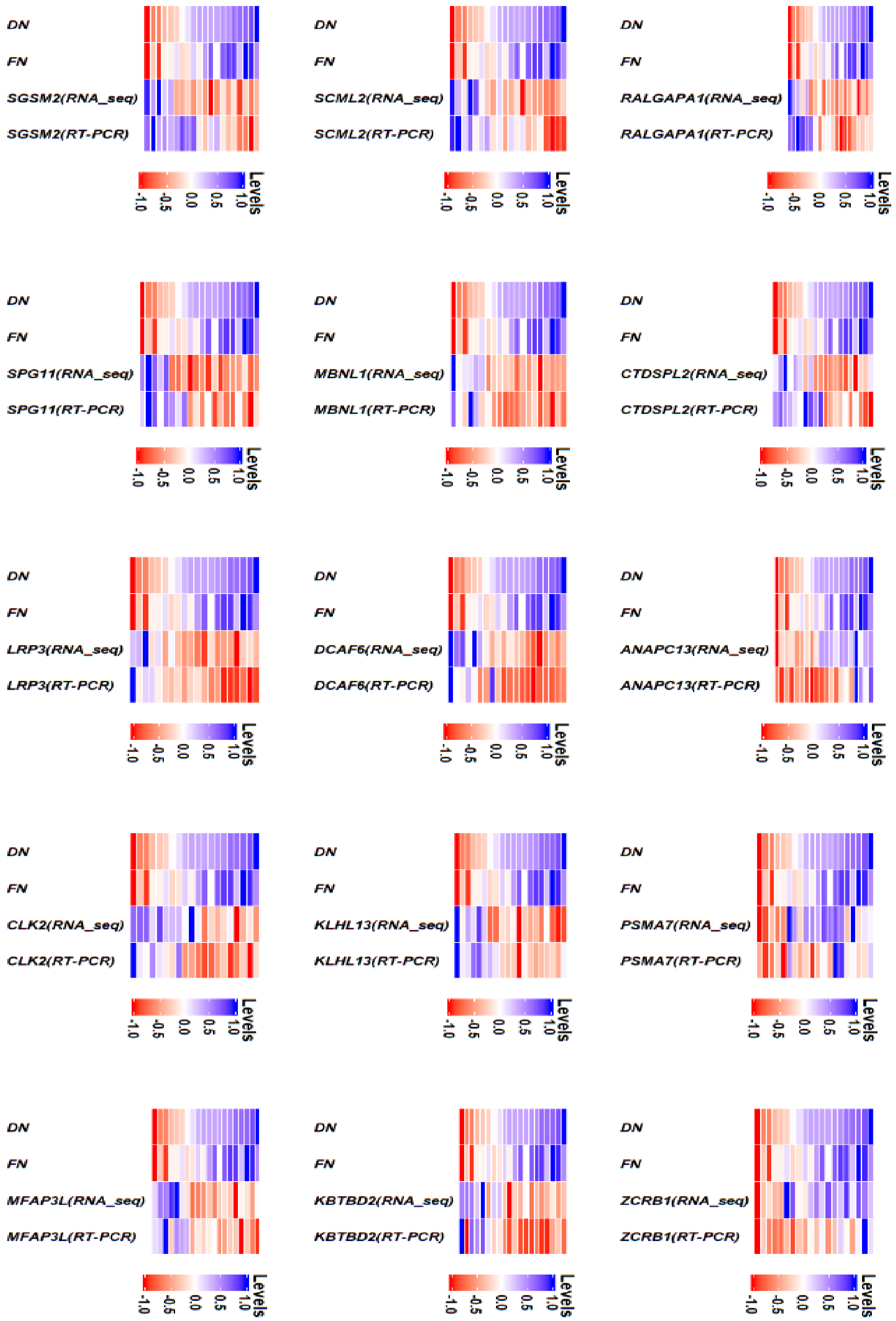

Figure S2 the heat maps of hub genes expression levels (including RNA seq and qPCR) and duration of fertility traits (including DN and FN). The expression levels of RNA-seq and qPCR of 20 samples, including their duration of fertility traits, were rescaled from -1 to 1 and plotted. DN = the number of days post-insemination until the last fertile egg was produced; and FN = the number of fertile eggs were produced after a single AI.
